# Safety of occasional ingestion of gluten in patients with celiac disease: a real-life study

**DOI:** 10.1186/s12916-020-1511-6

**Published:** 2020-03-16

**Authors:** Luca Elli, Karla Bascuñán, Lorenzo di Lernia, Maria Teresa Bardella, Luisa Doneda, Laura Soldati, Stefania Orlando, Francesca Ferretti, Vincenza Lombardo, Giulio Barigelletti, Alice Scricciolo, Sabrina Fabiano, Maurizio Vecchi, Leda Roncoroni

**Affiliations:** 1 Center for Prevention and Diagnosis of Celiac Disease, Gastroenterology and Endoscopy Unit, Department of Pathophysiology and Transplantation, Fondazione IRCCS Ca’ Granda Ospedale Maggiore Policlinico, University of Milan, Via F. Sforza 28, 20122 Milan, Italy; 2 Department of Nutrition, School of Medicine, University of Chile, Santiago, Chile; 3 Department of Health Sciences, University of Milan, Milan, Italy; 4 Department of Biomedical, Surgical and Dental Sciences, University of Milan, Milan, Italy; 5 Cancer Registry Unit, Fondazione IRCCS Istituto Nazionale dei Tumori, Milan, Italy

**Keywords:** Celiac disease, Gluten, Gluten-free diet

## Abstract

**Background:**

Gluten-free diet (GFD) decreases the quality of life of celiac disease (CD) patients, who frequently ask to occasionally ingest gluten-containing food. We evaluated CD patients reporting voluntary and occasional transgressions to their GFD.

**Methods:**

From October 2017 to September 2018, the patients reporting occasional and voluntary gluten ingestion (GFD-noncompliant) were prospectively enrolled. These patients underwent clinical examination, blood tests, duodenal biopsy, capsule enteroscopy (CE), and a validated food-frequency questionnaire (FFQ) assessing the frequency and quantity of gluten intake. Mortality was calculated and compared to the general population. A group of patients on strict GFD (GFD-adherent) acted as controls.

**Results:**

One thousand three hundred seventy-eight CD patients were evaluated during the study period. One hundred nine (8%) reported occasional (weekly or monthly) voluntary ingestion of gluten. The mean gluten intake was 185.2 ± 336.9 g/year, and the duration of their incorrect GFD was 8.6 ± 6.9 years. Among the noncompliant patients, 57% did not present any histological alteration; furthermore, the Marsh score profile was not different between compliant and noncompliant patients. Seventy percent did not present any alteration at CE. Seventy-five percent of patients reported no gastrointestinal symptoms after gluten ingestion. Twenty-three percent of patients in the GFD-noncompliant group presented positive tTG-IgA. No association was found between gluten intake, clinical symptoms, and biomarkers. Mortality was not different between the groups and the general population.

**Conclusions:**

Our results are that in a real-life scenario, a group of CD patients on long-term gluten intake showed no significant clinical symptoms or small bowel damage, thus suggesting that a degree of tolerance towards gluten consumption can be reached.

## Background

Celiac disease (CD) affects approximately 1% of the population worldwide [1] and is defined as an autoimmune enteropathy triggered by the ingestion of gluten proteins derived from wheat, rye, and barley [2]. CD is characterized by an impaired immune response in genetically susceptible individuals (carrying the HLA DQ2 and/or DQ8 haplotypes) and leads to the inflammation and atrophy of the small bowel (SB) mucosa [2]. As a consequence, the classical clinical picture of CD is characterized by nutrient malabsorption with several signs of malnutrition, but extra-intestinal symptoms and association with other autoimmune disorders are frequently present as signs of systemic disease [3, 4].

Nowadays, CD is considered a permanent condition of immunological intolerance to gluten, which requires strict lifelong gluten-free diet (GFD) [3]. The withdrawal of gluten from a patient’s diet usually results in the recovery of his/her intestinal mucosa with an improvement of symptoms, SB absorption, and normalization of the circulating autoantibody (anti-type 2 transglutaminase IgA, TG2) [5]. It is not clear which of these targets should be considered as the primary or secondary endpoint of GFD for CD patients towards their long-term prognosis [6]. Although safe and efficient, GFD is very restrictive, resulting as a burden on social life and quality of life (for both patients and caregivers) [7–10] and frequently with poor compliance [11–13]. Moreover, the clinical response to inadvertent or voluntary gluten ingestion by treated CD patients can be very heterogeneous, ranging from gastroenteritis-like pictures to no symptoms [3, 14]. Consequently, patients’ motivation or understanding in the following strict GFD is often poor. Moreover, it is not known if the prognosis of patients can be made worse by occasional gluten ingestion, especially in patients without symptoms [15].

This issue is crucial in clinical practice for the management of those CD patients that report occasional ingestion of gluten and whose number can reach up to 30% of cases [16].

Recent data has demonstrated that several CD patients can abandon GFD and maintain a healthy state with no adverse events or CD recurrence [17]. A large group of them will develop intestinal lesions after different exposure periods to gluten consumption, but the fact that some of them seem to tolerate gluten for long periods is intriguing [18]. Thus, the actual role of GFD in the prevention of CD-related complications (e.g., refractory disease, intestinal lymphoma) and other immune disorders is debated [15, 19].

The present work aimed to evaluate the dietary, clinical, endoscopic, and histological characteristics of patients with CD reporting occasional and voluntary transgression to GFD.

## Methods

### Patients

A prospective study was carried out from October 2017 to September 2018 at the “Center for Prevention and Diagnosis of Celiac Disease”, Fondazione IRCCS Ca’ Granda Ospedale Maggiore Policlinico in Milan (Italy). CD diagnosis was made according to the national and international guidelines [20, 21] including positivity to anti-type 2 transglutaminase IgA (TG2) and the presence of villous atrophy at duodenal histology, according to the Marsh-Oberhuber classification [22]. All the subjects referring to the outpatient service were clinically evaluated by an expert gastroenterologist, and they were interviewed by a trained nutritionist who evaluated the adherence of the patients to their GFD. Adherence to the GFD was evaluated by means of an in-depth interview executed by a nutritionist trained in the treatment and follow-up of patients affected by gluten-related disorders [23].

The patients were discriminated on the basis of the voluntary ingestion of gluten-containing food types. The kind of symptoms (bowel habits, abdominal pain, dyspepsia, meteorism, diarrhea, asthenia, constipation, gastro-esophageal reflux, etc.) arising after gluten ingestion was reported, when present. CD patients underwent upper endoscopy with duodenal biopsies. During gastroscopy (Pentax, EG27-i10 gastroscope), at least 4 oriented biopsies from the distal duodenum and 2 from the bulb were taken using standard endoscopic forceps (Boston Scientific, Radial Jaw™ 4) and were routinely processed for hematoxylin-eosin and CD3 staining as previously described [24]. All the obtained biopsies were reviewed by a pathologist and classified according to the Marsh-Oberhuber criteria [22]. Further, capsule enteroscopy (CE) was carried out after SB cleaning the day before the procedures with 2 L polyethylene glycol and overnight fasting. An axial view capsule system (Pillcam SB3, Medtronic) was used. At the end of the examination, the data recorded from the capsules were acquired. The recorder was removed after 12 h and the data downloaded to the dedicated system. CEs were read by an expert physician reading more than 100 CEs per year [25]. A suspicion of mucosal atrophy was posed in the presence of mosaicism, scalloping, granular mucosa, and/or loss of SB folds.

The histological and serological data from a randomly selected fully GFD-compliant, TG2 IgA-negative group of CD patients examined over the same period of time were used as control data.

The patients who agreed to participate gave their written informed consent and were enrolled in the study. The local Ethics Committee for Human Research of the City of Milan approved the study protocol (ref. no. 344_2018).

### Evaluation of gluten intake

For the patients reporting voluntary and occasional ingestion of gluten-containing food, the gluten consumption was assessed by means of a frequency food questionnaire (FFQ) validated and modified in the CD population [26]. FFQ was used to evaluate the frequency and the quantity of gluten-containing foods consumed during the last 12 months. A trained nutritionist administered the questionnaire: information on frequency, type of meals, and when gluten was consumed was collected. In addition to specific questions on foods with predefined gluten content, the FFQ also included open questions to detect and include any eventual (sporadic) gluten intake.

To understand the dietary source of gluten and which foods were most frequently consumed, a qualitative analysis of the diet was carried out; foods were grouped into five food categories: (a) bread and substitutes, (b) sweets, (c) beer, (d) pasta and other cereals, and (e) pizza/focaccia.

The intake of gluten from each food category was estimated on the basis of conversion factors (8.9 g gluten/100 g wheat flour, 4.2 g from barley, 3.0 g from rye, and 1.29 from oats) [27]. When analyzing beer consumption, the gluten content was obtained using a specific conversion factor, 0.00185 mg gluten/ml beer [28].

### Mortality

In order to avoid any bias on the mortality rate, death and causes of death were collected from the Central Register of Italy’s National Health Service collecting epidemiological data on the whole Italian population. The analysis was carried out on the overall mortality levels for all causes/pathologies. Comparisons were achieved using the mortality tables of Lombardy’s population (from Italy’s National Institute of Statistics, ISTAT). The SURVSOFT® V.2.0 software was used for the analysis (Cancer Register Bavaria, URL: http://www.krebsregister-bayern.de/software_e.html) [17]. The standardized mortality ratio (SMR) was defined as the ratio of the observed number of deaths in the population being studied and the expected number of deaths in a comparable group of individuals from the general population, matched with respect to the main factors affecting mortality, commonly age, sex, and calendar period.

### Statistical analysis

The data are described as mean ± SD or median (interquartile range) unless otherwise indicated. The continuous demographic variables were compared between the groups using independent Student’s *t* test. Fisher’s exact test was used to evaluate the distribution of categorical variables (i.e., gender distribution, the presence of gastrointestinal symptoms), the Marsh score between the groups of adherence to the GFD. STATA® rel. 13.1 (StataCorp, College Station, TX) was used, and statistical significance was set at 5% α level.

## Results

Among the 1378 adult CD subjects [mean age 48 ± 16, mean age at diagnosis 37 ± 34, 344 (25%) males] referred to our outpatient service during the study period, 109 (8%) [mean age 45 ± 13, mean age at diagnosis 29 ± 19, 38 (35%) males] reported some voluntary and occasional ingestion of gluten-containing food. The number of males was significantly (*p* = 0.03) higher in the group of patients referring to voluntary gluten ingestions. No differences in age or age at diagnosis were observed. Notably, the mortality of patients reporting some voluntary occasional ingestion of gluten was significantly lower when compared to the general population one (SIR 0, 95% CI 0–0.61).

**Fig. 1:**
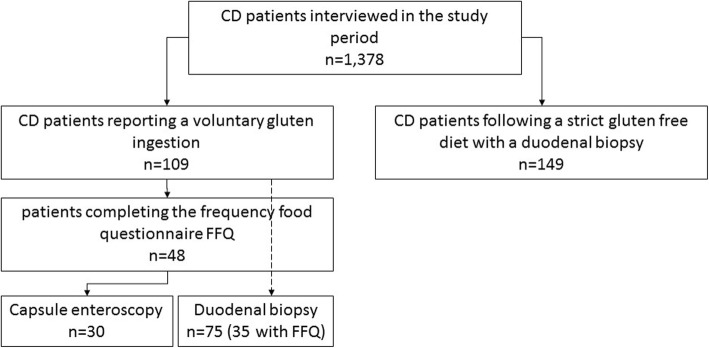
Flow chart on the patients participating in the study

**Fig. 2:**
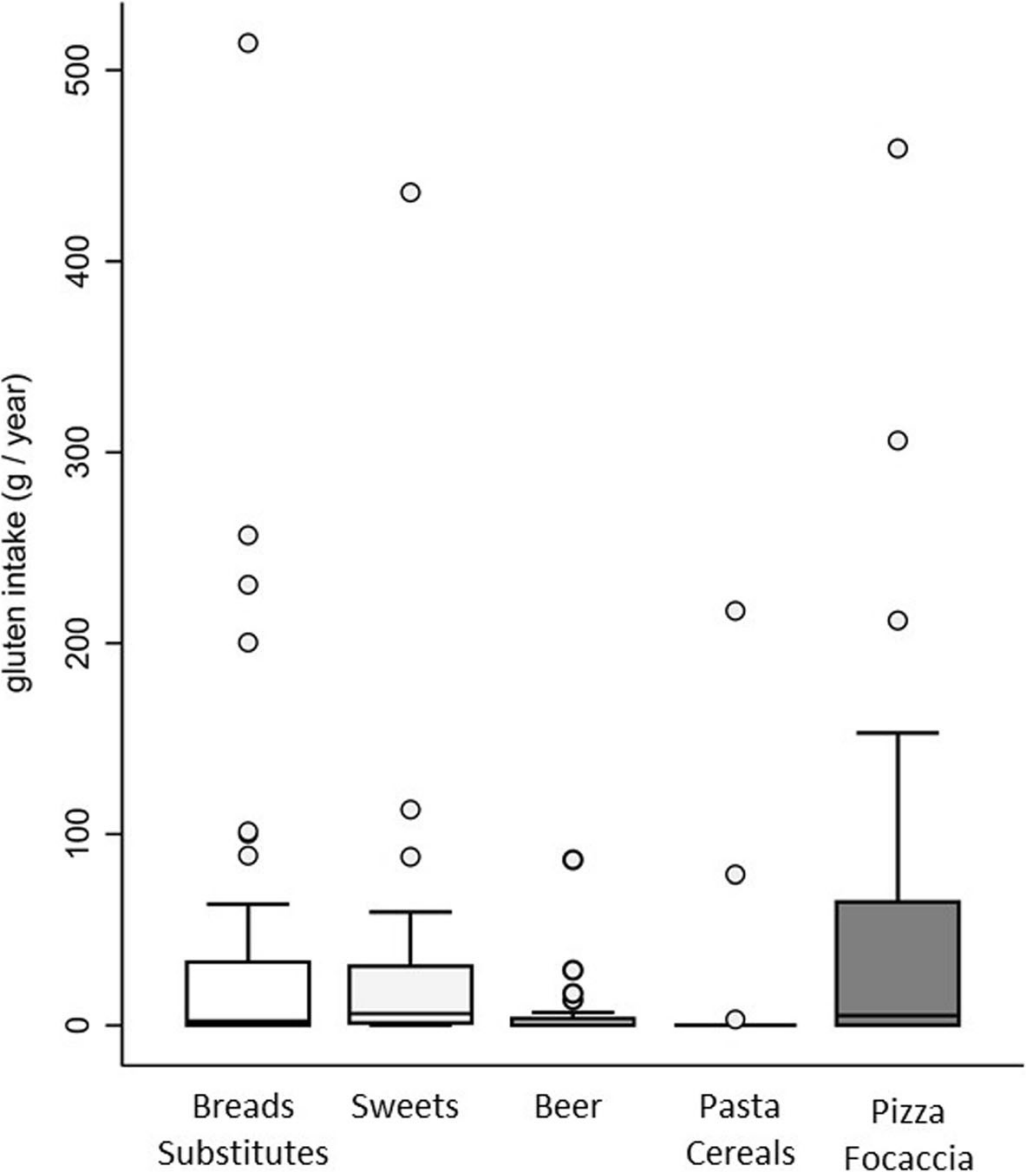
Gluten intake from the main alimentary groups

Among the patients reporting voluntary gluten ingestion, 48 (44%) patients completed a frequency food questionnaire (FFQ) to estimate the amount of gluten ingested per year; 149 patients following a strict GFD composed the control group (their demographic and clinical details are reported in Table 1). The complete study flowchart is provided in Fig. 1. In the GFD-noncompliant group, the mean amount of gluten ingested by patients was 185.2 ± 336.9 g/year (0.5 ± 0.9 g/day) for a mean period of 8.6 ± 6.9 years. The frequency of voluntary ingestion of gluten by the patients was usually once a week or month (4% of patients ingested gluten daily, 40% at least once a week, 35% on a monthly basis, and 21% at least once a year). The description of the alimentary sources of gluten in noncompliant patients is shown in Fig. 2. Dietary gluten mainly came from bread and its substitutes and from pizza/focaccia, pasta, and cereals. In the considered food groups, there were no differences in the intake of gluten. Among patients fulfilling the FFQ, 36 (75%) reported no symptoms after ingestion of gluten-containing food. In the 12 (25%) patients reporting a symptomatic relapse after voluntary gluten ingestion, the symptoms were change in bowel habits (16%), abdominal pain (25%), constipation (8%), meteorism (16%), dyspepsia (12%), asthenia (16%), and gastro-esophageal reflux (25%). This “symptomatic” group did not present any correlation in terms of tTG-IgA values or histology.

**Table 1:** Characteristics of enrolled patients

	GFD-noncompliant (*n* = 48)	GFD-adherent (*n* = 149)	*p* value
Age, years	45.5 ± 16.2	43.7 ± 12.5	0.357
Gender, M/F	17/31	21/128	0.001
Age at CD diagnosis, years^†^	30 (6.5–49.2)	33 (25–41)	0.431
Duration of the diet, years	8.6 ± 6.9	5.9 ± 7.6	0.110
Positive anti-transglutaminase antibody, %	22.9	0	0.000
Presence of autoimmune diseases, *n* (%)	14 (29%)	30 (20%)	0.23

^†^Data as median (interquartile range)

**Fig. 3:**
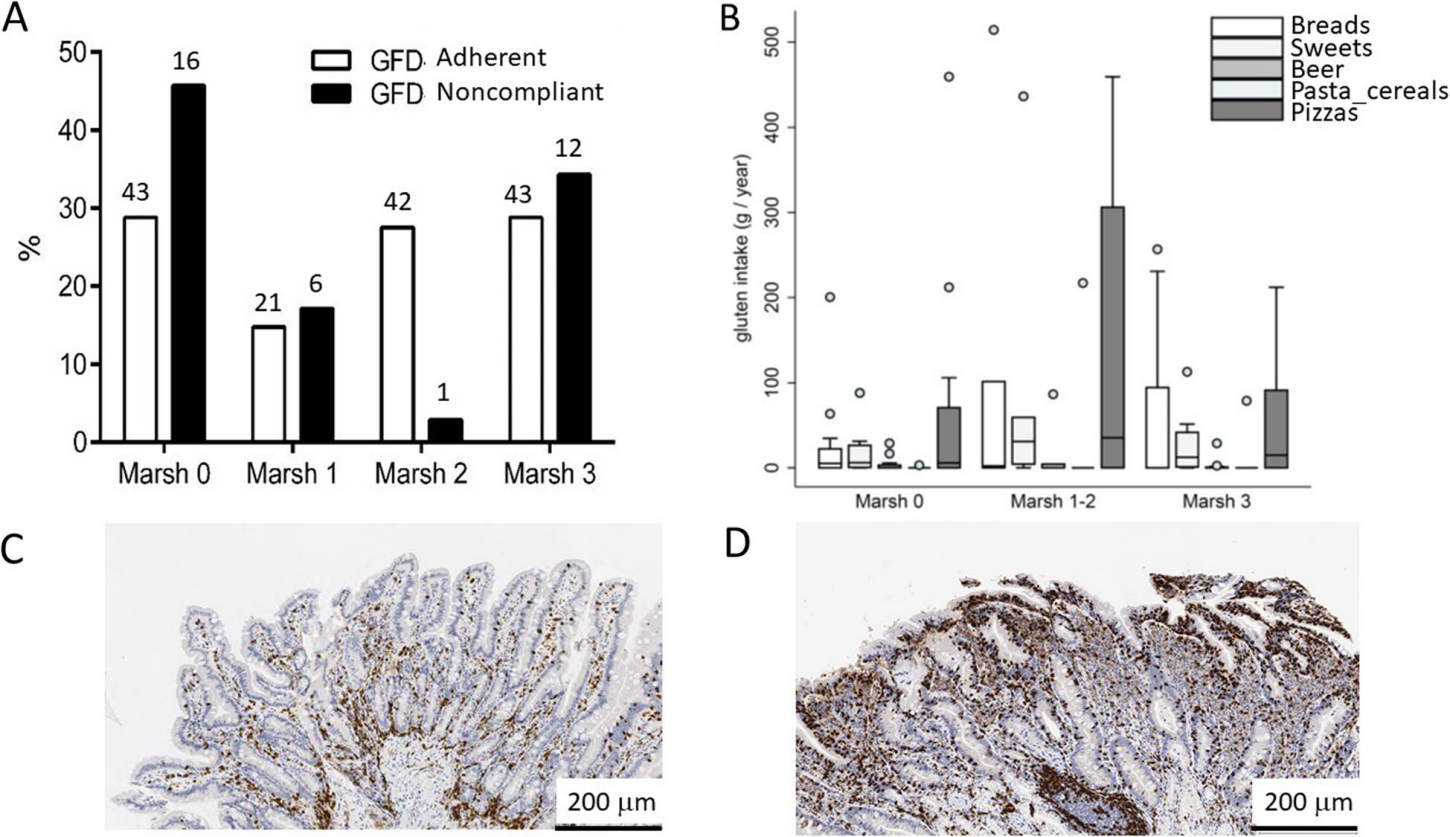
Distribution of patients (35 GFD-noncompliant vs. 149 GFD-adherent), according to the Marsh classification (**a**) and according to the sources of gluten (**b**). **c**, **d** Two examples of duodenal histology with CD3 staining of a noncompliant and a compliant patient, presenting a normal mucosa (Marsh 0) and severe (Marsh 3c) villous atrophy are reported

In the GFD-noncompliant, 22.9% had positive tTG-IgA values (Table 1). In general, 75 (69%) of noncompliant patients underwent upper endoscopy with duodenal histology; notably, 43 (57%) presented an unremarkable duodenal histology (Marsh 0); 50 (66%) presented normal or nonatrophic lesions (i.e., Marsh 0, 1, or 2), and 25 (33%) showed signs of atrophy, demonstrating a Marsh profile similar to the patients of the compliant group. Again, when only patients fulfilling the FFQ (35) have been considered, the Marsh score defining the absence of mucosal damage (Marsh 0) was evidenced in 43 (28.8%) patients in the GFD-adherent vs. 16 (45.7%) patients in the GFD-noncompliant group (*p* = 0.55). The same comparison for duodenal atrophy (Marsh 3) showed a 28.8% in the GFD-adherent compared to the 34.3% in the GFD-noncompliant group. In Fig. 3a, the different grades of villous atrophy are reported; no statistical differences have been found between the compliant and noncompliant groups. Moreover, no statistical differences were found when comparing the gluten intake of GFD-noncompliant patients with or without duodenal histologic damage: the noncompliant patients with Marsh 0 duodenal histology consumed 131.4 ± 154.4 g/year, Marsh 1–2 472.7 ± 707.9 g/year, and Marsh 3 187.9 ± 206.0 g/year. More details about the types of gluten-containing food ingested and their relationship to duodenal histology are reported in Fig. 3b. In Fig. 3c and d, two duodenal histologic images are reported. Moreover, there was no association between the frequency of gluten intake and intestinal mucosal damage (*p* = 0.896), gastrointestinal symptoms (*p* = 0.292), tTG-IgA positivity (*p* = 0.346), and the extent of the atrophic lesion as reported by CE (*p* = 0.209).

**Fig. 4:**
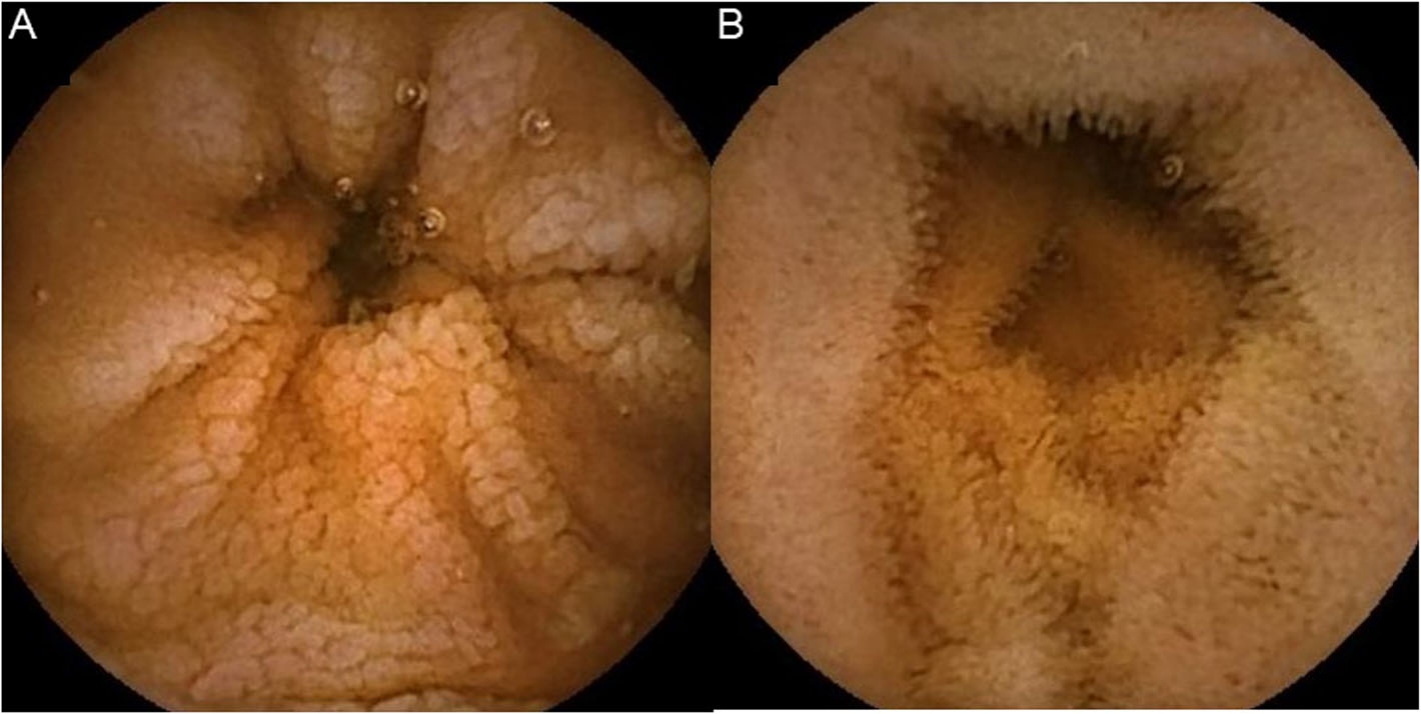
Capsule endoscopy investigations presenting atrophic (**a**) and normal (**b**) mucosa

Thirty (62%) noncompliant CD patients underwent CE, and 21 (70%) turned out unremarkable. In 9 (30%) cases, endoscopic signs of mucosal atrophy were described at CE. The following endoscopic signs of atrophy have been reported: mosaicism in 3 cases; mosaicism and scalloping in 3; scalloping and granular mucosa in 1; mosaicism, scalloping, and granular mucosa in 1; and granular mucosa in 1 case. The extension of SB atrophy expressed as the percentage of the SB transit time was 8.0 ± 3.2%. Again, the amount of gluten voluntarily ingested by patients with an atrophic mucosa at CE vs. CD patients without atrophy at CE was not statistically different, being 111.5 ± 159.7 and 211.9 ± 188.8 g gluten per year, respectively. Considering the presence of atrophy at histology as the reference standard (all patients undergoing CE were histologically evaluated), sensitivity and specificity of CE were 0.6 (95% CI 0.26–0.88) and 0.85 (95% CI 0.61–0.97), respectively. In Fig. 4a and b, two examples of CE investigations are represented.

## Discussion

Our study has shown that occasional and voluntary dietary gluten intake is not associated with the onset of clinical, serological, histologic, or endoscopic signs of CD for a group of CD patients. In particular, no association was found between histological alterations and the amount of gluten intake after CD diagnosis.

CD is the most common autoimmune enteropathy in the Western countries, and strict lifelong GFD is considered the only available treatment, usually inducing symptomatic remission, mucosal healing, and normalization of the serological alterations [3]. However, looking at GFD without a “dogmatic” point of view, a number of ambiguities and/or questions arise [15]: what is the prognosis of patients occasionally assuming gluten? What is the value of histology and serology during follow-up? Can some patients develop any tolerance to gluten? What is the role of GFD in asymptomatic patients? As a consequence, the “historical theory” that strict GFD is necessary for all CD patients and, forever, is somehow questionable. Furthermore, GFD usually worsens the quality of life of CD patients, and thus, a number of patients present a low degree of compliance to GFD because of their occasional ingestion of gluten, for example, during social events [5, 16].

In our cohort of noncompliant CD patients, mainly composed of asymptomatic patients, voluntary gluten ingestion has occurred on a weekly or monthly basis and has been maintained by the patients over a long period of time. During such time, those patients have been followed by our outpatient service on an annual basis through nutritional counseling, gastroenterological visits, and hematological and endoscopic investigations. Notably, the mortality of these patients is not different (if not lower) when compared to the mortality of the general population. The results from the studies to date investigating the mortality risk in CD patients are conflicting. The major problems in clearly defining such a risk come from the difficulty to distinguish between diagnosed and undiagnosed CD patients, patients with different CD sub-types, and to extrapolate those patients affected by refractory celiac disease (RCD) from the population database. RCD, although rare (less than 1% of CD patients), presents a very high risk of malignant transformation and consequently a high mortality rate: the presence of RCD in the analyzed cohorts could represent a bias in studies investigating mortality in CD [29, 30]. If some studies showed a fourfold increased mortality in CD [31, 32], mainly due to lymphoma, without demonstrating a clear connection with gluten exposure, often only indirectly suggested [33], other studies have failed to confirm this scenario [34]. In line with our findings, Olen et al. have analyzed mortality in GFD-compliant and GFD-noncompliant patients without finding any connections to the development of lymphoma [35].

Looking at the duodenal histology (usually considered as the reference standard for CD management) of noncompliant patients compared to the patients following a strict GFD, the incidence of patients maintaining duodenal atrophy was similar in the two groups (roughly 30%) without differences regarding the amount of ingested gluten between the noncompliant patients presenting or not any duodenal damage. It is well known that one third of CD patients correctly responding to GFD retain some sort of duodenal damage at histology [36]. The clinical significance of this finding is unknown and controversial. For this reason, it is doubtful if the duodenal damage found in noncompliant patients is really induced by gluten ingestion or by chance. Moreover, in order to investigate the presence of small bowel lesions beyond the Treitz ligament, as indicated by the recent guidelines and meta-analyses [37, 38], CE has been performed without showing any significant alterations but some signs of proximal atrophy in a small part of patients.

In our cohort of noncompliant patients, only the presence of circulating tTGA was significantly increased as compared to compliant controls. However, the tTGA levels do not correlate with the dose of ingested gluten or with the frequency of its ingestion. This finding is in line with earlier studies demonstrating the presence of tTGA in noncompliant patients, especially during childhood. Again, the prognostic significance of such peripheral signs in the absence of symptoms or histologic alterations remains unclear and not correlated to any malignancy development [30].

There is evidence supporting the hypothesis that it would be possible to achieve a certain level of tolerance to gluten after a variable period depending on each particular patient. In this regard, the systematic reviews available to date have focused on determining what can be considered as the highest safe intake of gluten for CD patients. Hischenhuber et al. have concluded that the maximum daily intake of gluten should be between 10 and 100 mg [39]. Other authors have reported that this limit is highly variable with some patients tolerating an average of 34–36 mg of gluten per day. Although there is no evidence to determine a single definitive threshold, a daily gluten intake of < 10 mg is unlikely to cause significant histological abnormalities in these patients [40]. This daily limit can represent an apparent individual tolerance level. It has been supposed that tolerance to gluten can be related to the age at CD diagnosis: individuals with diagnosis during childhood could develop a greater degree of tolerance compared to diagnosis at adulthood [41]. Other reports have demonstrated that some adult patients diagnosed during childhood did not show clinical or histological relapse after initially consuming dietary gluten [42, 43]. It should be mentioned that these studies came with methodological considerations mainly related to the evaluation of serology and histology of duodenal biopsies but not focusing on what could be the predictive factors of gluten tolerance. Notably, the studies investigating gluten tolerance or performing a gluten challenge administered gluten on a daily basis while the majority of patients in our study and in real life ingested gluten sporadically or only during specific events. This consideration suggests that it is the timing of the assumption (chronic vs. sporadic) more than the quantity of gluten that can drive a significant immunological and histological response.

Although our study involved a large number of patients followed over a long time period, it presents some points of weakness such as the absence of a randomized challenge with occasional gluten ingestion and the mono-centric design. Furthermore, we did not perform complete evaluations of comorbidities, apart from the presence of autoimmune diseases that appeared not related to gluten ingestion. This finding is in line with a previously published study analyzing the risk factors for the development of autoimmunity in CD [4].

## Conclusions

In summary, our study has shown that a group of celiac patients with long-term gluten intake shows no significant clinical symptoms or histological abnormalities, suggesting that a degree of tolerance towards gluten consumption can be reached. A better understanding of the mechanisms through which some patients can reach a degree of tolerance to gluten could indeed result in personalized GFD allowing for the intake of different amounts of gluten. This achievement can help to improve a patient’s quality of life and diminish the social burden associated with lifelong GFD.

## Data Availability

The datasets used and/or analyzed during the current study are available from the corresponding author on reasonable request.

## Abbreviations

CD: Celiac disease
CE: Capsule enteroscopy
FFQ: Frequency food questionnaire
GFD: Gluten-free diet
RCD: Refractory celiac disease
tTGA: Tissue transglutaminase antibodies
